# Management of a pediatric patient with rapidly progressive glomerulonephritis and cutaneous mucormycosis: a case report

**DOI:** 10.3389/fped.2025.1484145

**Published:** 2025-05-08

**Authors:** Lili Jia, Kaili Shi, Xiaoyi Sun, Feng Xu, Tao Sun, Chunlin Gao

**Affiliations:** ^1^ Department of Pediatrics, Jinling Hospital, School of Medicine, Nanjing University, Nanjing, China; ^2^ Department of Pediatrics, Jinling School of Clinical Medicine, Nanjing Medical University, Nanjing, Jiangsu, China; ^3^ National Clinical Research Center for Kidney Diseases, Jinling Hospital, Nanjing, China

**Keywords:** rapidly progressive glomerulonephritis, cutaneous mucormycosis, pediatric, management, compromised immune function

## Abstract

Mucormycosis is a highly invasive and rare opportunistic infection caused by mucor fungi, characterized by challenging diagnosis and rapid disease progression. It predominantly affects patients with compromised immune systems due to various reasons, such as kidney failure, long-term use of antibiotics or corticosteroids. We recently successfully treated a pediatric patient with rapidly progressive glomerulonephritis accompanied by severe cutaneous mucormycosis. To our knowledge, this is the first reported case of rapidly progressive glomerulonephritis nephritis accompanied by dermatophytosis in a pediatric patient. In this case, we share our management experience, including special nursing experience. Cutaneous mucormycosis progresses quickly and is difficult to diagnose and treat, especially in children with compromised immune function, warranting high vigilance from clinicians and nursing staff. Early diagnosis and targeted treatment are crucial for improving the prognosis of patients. Therefore, once there is a suspicion of a mucormycosis infection, we recommend the early application of various testing methods such as fungal culture, skin biopsy and genetic testing in order to to promptly confirm the diagnosis.

## Introduction

Rapidly progressive glomerulonephritis is a disease characterized by acute nephritic syndrome with rapidly deteriorating kidney function, and its pathological features are segmental necrotizing nephritis with crescent formation. Treatment options include high-dose corticosteroids and immunosuppressive therapy (1). Mucormycosis is a highly invasive and rare opportunistic infection caused by mucor fungi, characterized by challenging diagnosis, rapid disease progression, and a mortality rate exceeding 32%∼70% (2, 3). It predominantly affects patients with compromised immune systems due to various reasons, such as kidney failure, long-term use of antibiotics or corticosteroids, chemotherapy, or diabetic ketoacidosis (4). Cutaneous mucormycosis is one of common clinical presentations of mucormycosis. The initial form of infection may be induration or blisters, pustules, nodules, and necrotic ulcers (5).

We recently successfully treated a pediatric patient with rapidly progressive glomerulonephritis accompanied by severe cutaneous mucormycosis. The purpose of this study is to share our management experience with children suffering from cutaneous mucormycosis, in hopes of improving the prognosis for these patients.

## Case report

**Figure 1:**
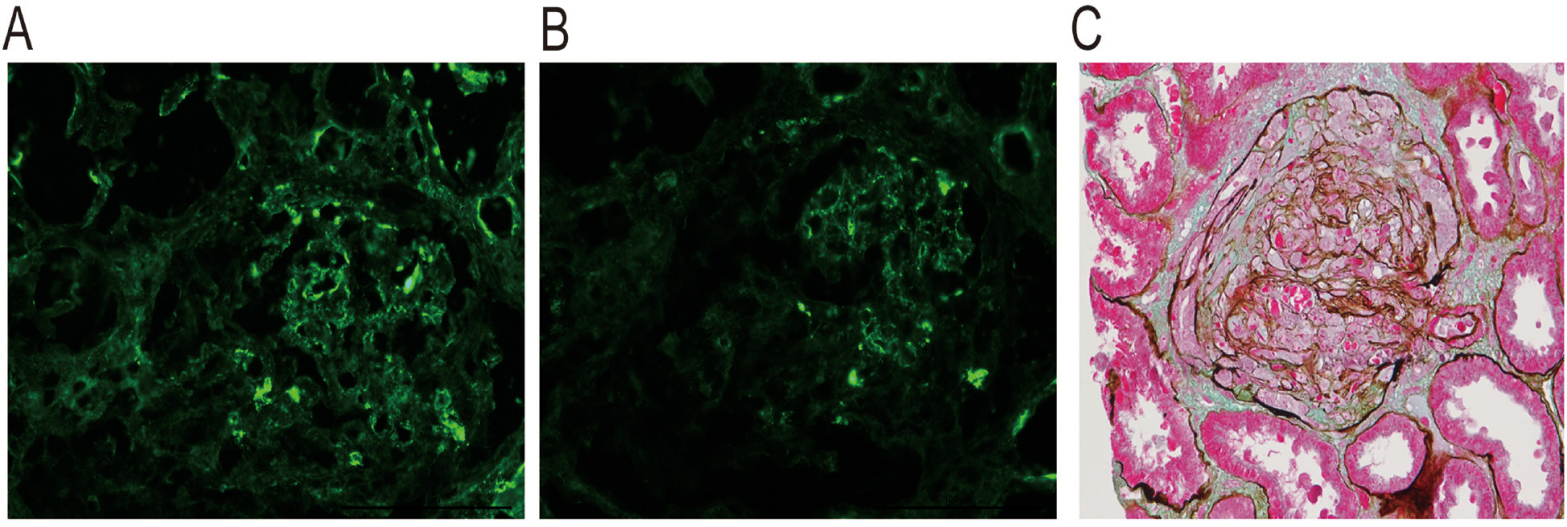
Kidney biopsy pathology of this patient. Immunofluorescence: **(A)** Granular deposition of C3 in the mesangial area; **(B)** Granular deposition of IgA in the mesangial area and vascular pole. **(C)** PAS-Masson stain was positive, and occasional eosinophilic materials seen in the mesangial area of glomerulus. This figure was provided by National Clinical Research Center of Kidney Disease, Jinling Hospital.

A 10-year-old female patient initially presented with gross hematuria and edema. She treated high-dose methylprednisolone pulse therapy and tacrolimus for immunosuppression in other hospital. Due to progressive kidney insufficiency and reduced urine output, she was transferred to our hospital. The girl was clinically diagnosed with rapidly progressive glomerulonephritis. Her blood and urine tests showed: serum creatine (SCr)-701.1 μmol/L, plasma albumin9 (Alb)-21.6 g/L, hemoglobin (Hb) −72 g/L, urine protein-++++, urinary red blood cell-5,028/ul, autoimmune antibodies (-). This girl underwent a kidney biopsy, and the pathological results showed glomerular crescent formation (86.67%) and global sclerosis (6.67%). PAS-Masson staining was positive, with visible crescent formation. kidney tissue immunofluorescence showed IgA deposition++ and C3 deposition++ (Figure 1). We administered continuous kidney replacement therapy (CRRT), infusion of human albumin and red blood cell suspensions for symptomatic supportive treatment.

**Figure 2:**
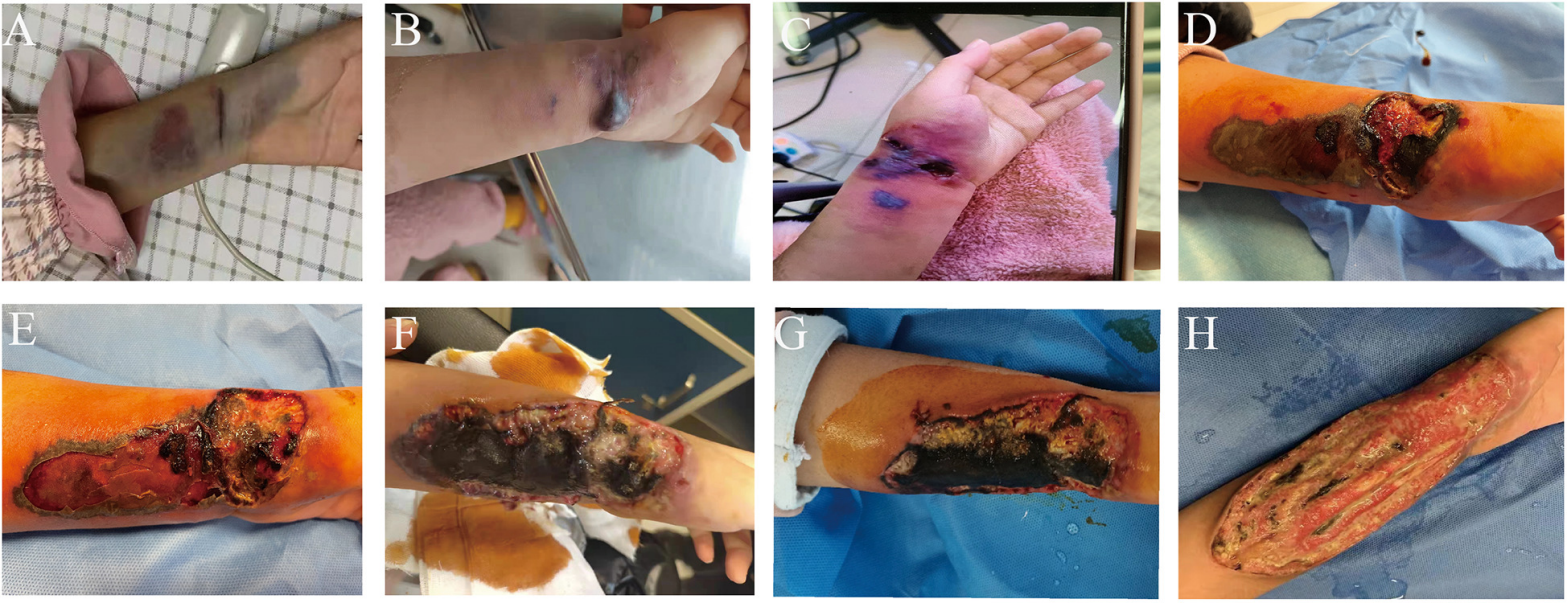
The evolution process of the skin condition on the patient's forearm. **(A)** Day 1; **(B)** Day 45; **(C)** Day 47; **(D)** Day 49; **(E)** Day 52; **(F)** Day 55; **(G)** Before the surgery; **(H)** After the surgery.

Upon admission, the patient's physical examination revealed a notable subcutaneous ecchymosis measuring 12 cm by 4 cm on the left wrist (Figure 2). In response to this finding, treatment was initiated with wet dressings of magnesium sulfate and topical mucopolysaccharide polysulfate cream. The ecchymosis showed significant reduction. On the 38th day post-admission, the patient reported a marked increase in pain in the left wrist. The physical examination revealed erythema with indistinct borders, mild edema, slightly elevated temperature, and no signs of skin damage, which are suggestive of phlebitis or cellulitis. To address these symptoms, Piperacillin-tazobactam was administered intravenously, alongside treatment with infrared irradiation and iodine wet dressings, aimed at enhancing blood circulation, reducing inflammation, and alleviating pain. However, on day 45, the patient experienced exacerbated pain in the left wrist along with the emergence of a 2 cm by 1 cm blister containing colorless, transparent fluid. The blister was aspirated and disinfected, followed by local dressing. Unfortunately, the patient's skin damage progressively worsened, resulting in the formation of localized ulcers and necrosis. On day 47, the wound area had expanded to approximately 2.5 cm × 1.5 cm, yet a smear of the wound secretion showed no abnormalities. On day 49, the patient's ulcer and necrotic wound exhibited further expansion, progressing to the left forearm, and presented with black eschar and multiple blisters. In response, we administered Cefperazone-Sulbactam treatment, local debridement, and physiotherapy. Nevertheless, satisfactory results were not achieved. On day 52, the wound increased in size by approximately 3 cm by 8 cm, deepening and extending into the muscular layer. By day 55, the wound revealed a large, deep ulcer measuring approximately 13 cm by 5 cm. The majority of the surface was covered with black eschar, and white exudate was observable. A second smear of the wound secretion and skin tissue biopsy were conducted, revealing the growth of mold hyphae and spores. Meanwhile, we performed next-generation sequencing (NGS) of the patient's secretions, which indicated a positive result for Mucor fungus.

On day 58, the patient underwent debridement of the skin wound under general anesthesia, along with limb vascular ligation. At the same time, the patient was treated with intravenous liposomal amphotericin B. Daily wound cleaning and dressing changes were followed by local application of amphotericin B antifungal and recombinant human basic fibroblast growth factor to promote wound healing. Due to gastrointestinal intolerance symptoms such as nausea and vomiting, the patient was switched to oral posaconazole sequential therapy. After the surgery, the skin infection gradually came under control. As the child's kidney function had not improved and other potential infections were ruled out, the child underwent peritoneal dialysis catheter placement surgery. Post-surgery, peritoneal dialysis was conducted as a substitute treatment. During follow-up, we observed that the wound on the left forearm healed well, showing fresh tissue growth, and multiple microbial cultures were negative. At the 6-month follow-up, the patient's forearm skin tissue was gradually growing, and by the 9-month follow-up, the skin tissue had fully healed. The patient was regularly undergoing peritoneal dialysis treatment and awaiting a kidney transplant. Unfortunately, she passed away due to severe lung infection accompanied by respiratory failure caused by COVID-19, and thus, we were unable to obtain a photo of the patient's completely recovered skin.

## Discussion

Mucormycosis is highly invasive infection with a poor prognosis (6). It is a rare opportunistic infection and the most common pathogens causing it are from the Rhizopus spp, Mucor spp, and Lichtheimia spp, followed by Rhizomucor spp, Cunninghamella spp, Apophysomyces spp, and Saksenaea spp (7, 8). It typically occurres in patients with compromised immune systems due to various reasons, such as kidney failure, prolonged use of antibiotics or corticosteroids (4). In recent years, with the outbreak of COVID-19 globally, cases of concurrent mucormycosis have not been uncommon (9). As of now, there are no reported cases of pediatric patient with crescentic glomerulonephritis complicated by mucormycosis.

We analyzed the possible causes of the acquired mucormycosis infection in this case. Our patient was diagnosed with crescentic glomerulonephritis and received high-dose methylprednisolone treatment along with regular doses of corticosteroids and immunosuppressants like tacrolimus. Additionally, chronic severe proteinuria leading to hypoalbuminemia and severe kidney dysfunction resulted in the child's weakened immune system, creating an opportunity for pathogenic microbial invasion. It is important to highlight that following only one arterial blood gas analysis and the aspiration of a blister, the patient developed skin lesions that progressively advanced to ulceration and necrosis. Despite strict adherence to disinfection protocols, the compromised skin provided an opportunity for fungal invasion. We believe that maintaining skin integrity is crucial in preventing fungal infections, particularly in critically ill patients. The presence of broken skin can serve as a gateway for pathogens to enter, which is a risk factor that warrants special attention in clinical practice.

Unfortunately, this patient ultimately passed away due to COVID-19, which further sounded an alarm for us. Long-term use of immunosuppressive therapy greatly increased the possibility of potential infections in children who suffered kidney diseases. For such patients, we need to closely monitor and pay more attention to the standardization of invasive procedures.

In this case, the patient's skin damage progressed rapidly, from mild pain to extensive skin necrosis in just two weeks. Early in the appearance of skin symptoms, we conducted pathogenic microbial tests, but failed to detect the pathogen. Subsequently, a skin biopsy revealed the growth of mold hyphae and spores, and NGS confirmed Mucor fungus infection. The study of Maria Ziaka et al. showed that only 11.5% of pediatric patients were diagnosed only through fungal culture (3). The research suggested that the combination of multiple diagnostic methods can increase the detection rate of the pathogen in patients. This suggests that cutaneous mucormycosis progresses quickly and is difficult to diagnose and treat, especially in children with compromised immune function, warranting high vigilance from clinicians and nursing staff. Early diagnosis and targeted treatment are crucial for improving the prognosis of patients.

According to the the European Confederation of Medical Mycology (ECMM), When mucormycosis is suspected, it is necessary to actively remove the infected site surgically in combination with drug therapy (10). Liposomal amphotericin B 5–10 mg/kg per day is the first-line treatment for mucormycosis, followed by itraconazole and posaconazole as a secondary option (10–12). After confirming the diagnosis, we administered amphotericin B treatment. However, due to severe gastrointestinal reactions, it was necessary to switch to posaconazole.

In our patient, the skin lesions eventually underwent resolution, which was not only due to timely diagnosis and early medication but also closely related to our meticulous care. Here, we share some of our experiences regarding the treatment of such lessions. The routine skin dressing changes were not enough effective to this patient, and the previous method of using an outer layer to fix the dressing reduced the concentration of the medication, making it difficult to inhibit the growth of mucor fungus. We improved the dressing change method: after daily surgical disinfection and debridement, recombinant human basic fibroblast growth factor was sprayed on the wound, followed by covering the wound completely with sterile gauze soaked in amphotericin B solution (15 mg/20 ml), allowing full absorption of the medication. After 30 min, the gauze was discarded and a light layer of sterile gauze was applied. Topical application of amphotericin B can reduce lesion secretions and clear dormant spores around necrotic tissue in a short time. Additionally, children with kidney disease often have a long duration of illness and high costs, leading to significant psychological burden for the child and their parents. Active guidance to the child is also crucial in promoting recovery from the disease.

In conclusion, cutaneous mucormycosis progresses quickly and is difficult to diagnose and treat, especially in children with compromised immune function, warranting high vigilance from clinicians and nursing staff. Early diagnosis, targeted nursing and treatment are crucial for improving the prognosis of patients.

## Data Availability

The original contributions presented in the study are included in the article/Supplementary Material, further inquiries can be directed to the corresponding author.
